# The status of maternal and newborn health care services in Zanzibar

**DOI:** 10.1186/s12884-016-0928-6

**Published:** 2016-06-03

**Authors:** Bakar Fakih, Azzah A. S. Nofly, Ali O. Ali, Abdallah Mkopi, Ali Hassan, Ali M. Ali, Kate Ramsey, Theopista John Kabuteni, Godfrey Mbaruku, Mwifadhi Mrisho

**Affiliations:** Ifakara Health Institute, P.O. Box 78373, Dar es Salaam, Tanzania; Intergrated Reproductive and Child Health Program - Zanzibar Ministry of Health, P.O Box 236, Zanzibar, Tanzania; Columbia University Mailman School of Public Health, 722 W 168th St, New York, NY 10032 USA; Ministry of Health, Health Sector Program Support, DANIDA-Zanzibar, P.O.Box 236, Zanzibar, Tanzania; Maternal, Newborn and Child Health, World Health Organisation, P.O.Box 9292, Dar es Salaam, Tanzania

**Keywords:** Zanzibar, Emergency obstetric and newborn care, Signal functions

## Abstract

**Background:**

It is estimated that 287,000 women worldwide die annually from pregnancy and childbirth-related conditions, and 6.9 million under-five children die each year, of which about 3 million are newborns. Most of these deaths occur in sub-Saharan Africa. The maternal health situation in Tanzania mainland and Zanzibar is similar to other sub-Saharan countries. This study assessed the availability, accessibility and quality of emergency obstetric care services and essential resources available for maternal and child health services in Zanzibar.

**Methods:**

From October and November 2012, a cross-sectional health facility survey was conducted in 79 health facilities in Zanzibar. The health facility tools developed by the Averting Maternal Death and Disability program were adapted for local use.

**Results:**

Only 7.6 % of the health facilities qualified as functioning basic EmONC (Emergency Obstetric and Neonatal Care) facilities and 9 % were comprehensive EmONC facilities. Twenty-eight percent were partially performing basic EmONC and the remaining 55.7 % were not providing EmONC. Neonatal resuscitation was performed in 80 % of the hospitals and only 17.4 % of the other health facilities that were surveyed. Based on World Health Organisation (WHO) criteria, the study revealed a gap of 20 % for minimum provision of EmONC facilities per 500,000 population. The met need at national level (proportion of women with major direct obstetric complications treated in a health facility providing EmONC) was only 33.1 % in the 12 months preceding the survey. The study found that there was limited availability of human resources in all visited health facilities, particularly for the higher cadres, as per Zanzibar minimum staff requirements.

**Conclusion:**

There is a need to strengthen human resource capacity at primary health facilities through training of health care providers to improve EmONC services, as well as provision of necessary equipment and supplies to reduce workload at the higher referral health facilities and increase geographic access.

## Background

Despite the high priority given to maternal and child health, it is now clear that the realization of Millennium Development Goals (MDGs) by 2015 is unrealistic. Annually, it is estimated that 287,000 women worldwide die from pregnancy and childbirth-related conditions, and 6.9 million under-five children die each year, of which about 3 million are newborns. Most of these deaths occur in sub-Saharan Africa [1, 2].

The maternal health situation in Tanzania mainland and Zanzibar is similar to other sub-Saharan countries [3]. A significant proportion of maternal deaths occur as a result of direct and indirect obstetric complications between the third trimester and the first week after delivery [4–7]. Since many of the obstetric complications cannot be predicted, skilled delivery and access to emergency obstetric care continue to be the most important strategies to reduce maternal and perinatal mortality [8–14].

In Tanzania, estimates of maternal mortality vary based on the sources, but all have wide confidence intervals. For example the Tanzania Demographic Health Survey, (TDHS) [3] estimates 454 maternal deaths per 100,000 live births [3] while the UN estimates 790 maternal deaths per 100,000 live births in the same year [15]. Although separate estimates for Zanzibar are not available from the TDHS, a study of facility-based maternal deaths from 2005 to 2007 in Zanzibar showed a surprisingly high maternal mortality ratio among women who delivered in facilities [16]. Significant declines in child mortality have been observed in the Tanzania Mainland and Zanzibar, but with insufficient progress in reducing neonatal deaths [3, 17].

In committing to MDGs 4 and 5, the Government agreed to reduce the under-five mortality rate by two-thirds and reduce the maternal mortality ratio by three-quarters, by 2015 from 1990 levels. Many countries have been embarking on Emergency Obstetric and Newborn Care (EmONC) needs assessments in order to obtain the data necessary to improve maternal health services [18, 19]. As part of the government of Zanzibar’s efforts to improve maternal and newborn care, we undertook an assessment of the availability, accessibility and quality of maternal and newborn services in public and private health facilities in Zanzibar.

## Methods

### Design and Study area

A cross-sectional health facility survey was conducted in Unguja and Pemba Islands of Zanzibar between October and November, 2012.

### Description of the facilities

The study involved public, private for profit, and non-governmental organization health facilities providing maternal and child health (MCH) services. There were 224 health facilities in Zanzibar at the time of the survey. Sixty four percent of these facilities were government owned; 34 % were privately owned, while 2 % were parastatal. The health sector in Zanzibar includes three levels of care and corresponding facilities as follows: a) Primary level: Health Care Units and Centres (PHCUs, PHCU+ and Primary Health Care Centres-PHCCs) b) Secondary level: District Hospitals c) Tertiary level: Mnazi Mmoja National Hospital. PHCUs provide Primary health care services, PHCU+ are selected to provide additional services such as delivery, dental, laboratory and pharmacy services. PHCCs provide the same services as PHCU+ with the addition of inpatient and X-ray services. District hospitals provide second line referral services, including basic surgery and the tertiary hospital (Mnazi Mmoja Hospital) provides referral services.

### Sample size

A measure of relative variance was applied to determine the sample number of health facilities required [20]. All secondary and tertiary health facilities (43 facilities) offer maternity services and thus were included in the sample. A stratified random sampling procedure was applied to sample first line PHCUs. The targeted health facilities were divided into two strata. The first stratum was comprised of first line Primary Health Care Units (PHCUs) and the second stratum of PHCU+ and PHCC facilities.

All higher secondary and tertiary health facilities (43 facilities) were included in the sample as they offer maternity health services. The random sampling procedure was applied to get the remaining sample of the first line PHCUs. The sampling frame included 100 PHCUs in all districts. STATA 12.0 software was used to conduct random sampling required sample size (38 of 100 PHCUs). Our initial sample was 80 health facilities; however, one health facility was dropped due to unavailability of staff to participate in the study.

### Procedure

The assessment methods and modules developed by the Averting Maternal Death and Disability (AMDD) program (www.mailman.columbia.edu/research/averting-maternal-death-and-disability-amdd) at Columbia University, New York, USA, and United Nations (UN) Partners were adapted for local use. Methods included key informant interviews, observations, and data extraction. A workshop with the Zanzibar Ministry of Health (ZMOH) and other stakeholders was held to make the relevant adaptations to the Zanzibar context [21]. In order to ensure that the wording of the questions was correct and understood by both, interviewer and interviewee, study instruments were translated into Swahili and pilot-tested in Kivunge, Chukwani, Kitope, Jambiani and Mwera health facilities.

A total of 5 teams, each of which included a supervisor, data entry clerk and three research assistants (RAs), were recruited, ensuring at least one midwife per team. All interviewers had completed at least secondary school education. Research assistants and supervisors were trained for three days to ensure that they understood the objectives of the survey, the study methods and the content of the modules. During the training, roles of supervisors, research assistants and other study team members were also explained. A training manual was prepared to enhance the effectiveness of training and was also used as a reference for data collectors during the survey.

Teams were assigned to move around the districts ensuring data collection from all sampled facilities. With exception of hospitals, data collection required approximately one day per health facility. Supervisors contacted the health facility in-charge, assigned work to RAs, maintained continuous progress in data collection and reviewed the completed questionnaires for errors and omissions on a daily basis. Interviews were conducted with health facility in-charges or midwives at the PHCUs and PHCUs + and in-charges of the maternity wards, pharmacy, laboratory and human resource for PHCC and hospital levels.

### Data analysis

Data cleaning and consistency checks were conducted after the survey. Data were analyzed using STATA (version 12.0, College Station, Texas, USA). Analyses were conducted in accordance with UN guidelines for monitoring obstetric services [22], including performance of signal functions and the calculation of the EmONC indicators.

The World Health Organisation (WHO) handbook on monitoring emergency obstetric and neonatal care EmONC defines the signal functions as 1) administration of parenteral antibiotics, 2) administration of parenteral uterotonics, 3) administration of parenteral anticonvulsants; 4) manual removal of the placenta (MRP); 5) removal of retained products; 6) Assisted Vaginal Delivery (AVD); 7) neonatal resuscitation; 8) blood transfusion; and 9) obstetric surgery. The handbook classifies health facilities that have performed the first seven signal functions in the last three months as basic EmONC (BEmONC) facilities and those providing all nine signal functions are classified as comprehensive EmONC (CEmONC) facilities [22]. Furthermore, any facility providing at least one of the first seven signal functions was considered as partially functioning. A non-EmONC facility was defined as a facility which never provided any of the seven basic signal functions in the three-month period before the assessment.

## Results

### Availability of EmONC services

The availability of EmONC services was measured by determining the number of facilities that performed the complete set of required signal functions in the three-month period prior to the assessment. Only 7.6 % (6/79) of the visited health facilities qualified as fully functioning Basic Emergency Obstetric and Neonatal Care (BEmONC) facilities and 9 % (7/79) were comprehensive EmONC facilities. Twenty eight percent (22/79) of the visited health facilities were classified as partially performing BEmONC and 55.7 % (44/79) were considered non-EmONC facilities (Table 1).

**Table 1:** Facilities with Comprehensive Emergency Obstetric Neonatal Care (CEmONC) & BEmONC (*N* = 79)

	Health facility status			
	CEmONC % (*n*)	BEmONC % (*n*)	Partially BEmONC % (*n*)	Non-EmONC % (*n*)
OVERALL	9	7.6	27.9	55.7
Number of health facilities	7	6	22	44
Health facility type				
National Hospital	1.3 (1)	0 (0)	0 % (0)	0 (0)
District hospital	3.8 (3)	0 (0)	0 (0)	0 (0)
Cottage Hospital (PHCC)	1.3 (1)	2.5 (2)	1.3 (1)	0 (0)
PHCU+	0 (0)	5.1 (4)	21.5 (17)	16.5 (13)
PHCU	0 (0)	0 (0)	5.1 (4)	39.2 (31)
Maternity hospital	1.3 (1)	0 (0)	0 (0)	0 (0)
Private hospital	1.3 (1)	0 (0)	0 (0)	0 (0)

**Fig. 1:**
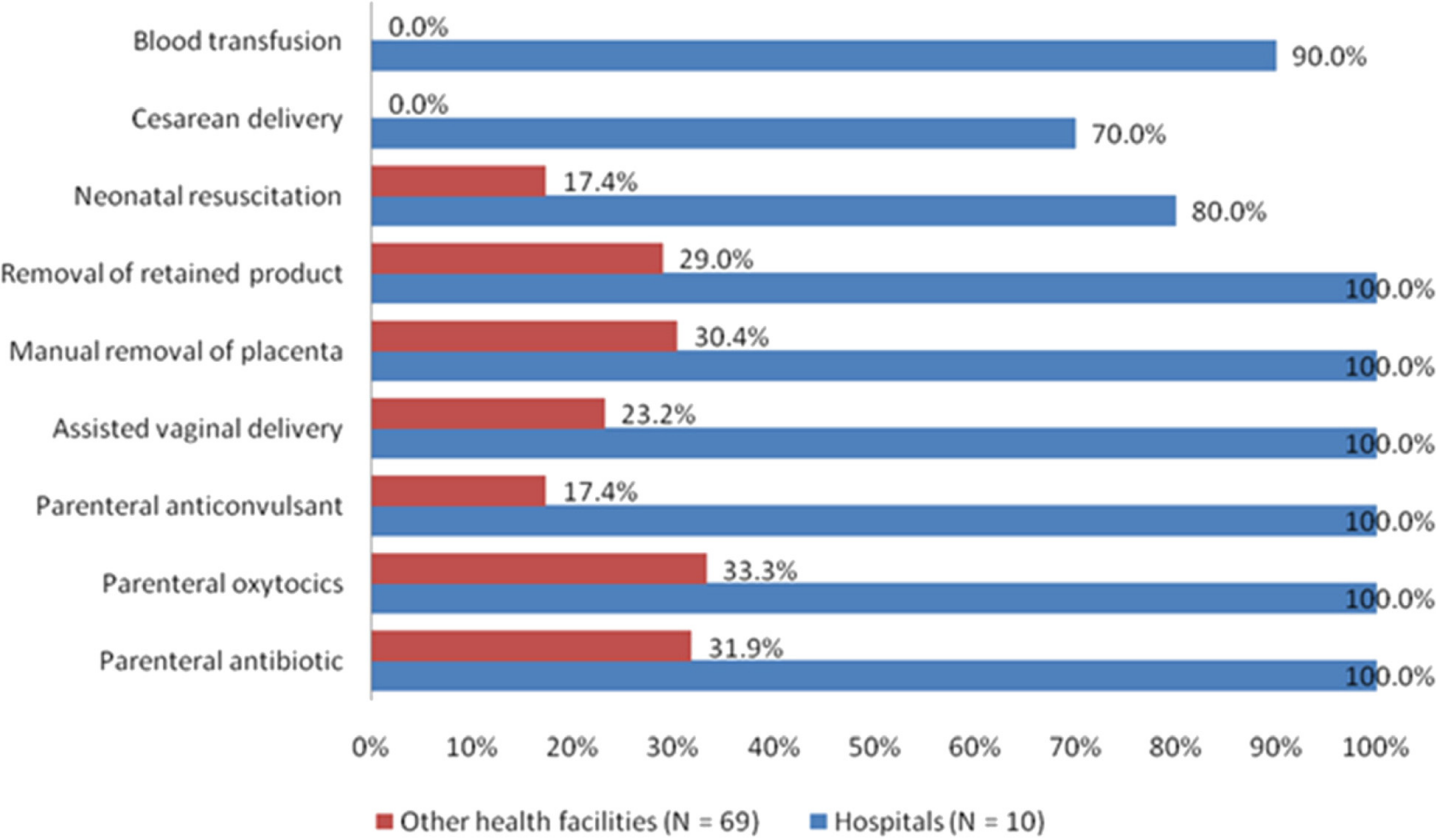
Proportion of health facilities in which each signal function was performed during the past 3 months, Zanzibar 2012

Figure 1 below shows the distribution pattern of health facilities in which each of the 7 signal functions were performed during the 3 months prior to the survey.

The WHO handbook on monitoring emergency obstetric care recommends the minimum acceptable number of EmONC facilities as 5 EmONC with at least one CEmONC per 500,000 population [22]. According to these criteria there are 20 % fewer EmONC facilities in Zanzibar than required. Yet, the number of CEmONC facilities surpassed the required minimum (133 %) (Table 2). Overall, minimum recommended level of EmONC services in Zanzibar has been met by 87 %.

**Table 2:** Distribution of Basic and Comprehensive EmONC in visited districts (*N* = 79)

	Total population*	Surveyed facilities (all)	Existing Basic EmOC	Existing Comprehensive	Min number of comprehensive required	overall minimum number of facilities required (Basic + Comprehensive)^a^	% of the facility available (Basic + Comprehensive)	% of comprehensive facility available
OVERALL	1460987	79	6	7	3	15	87	233.3
By Zones								
Pemba	461417	33	4	3	1	5	140	300
Unguja	999570	46	2	4	2	10	60	200
By district								
ChakeChake	104461	7	1	1	1	5	40	100
Mkoani	115228	9	1	1	1	5	40	100
Wete	125907	9	0	1	1	5	20	100
Micheweni	115820	8	2	0	1	5	40	0
Kaskazini A	106972	7	1	0	1	5	20	0
Kaskazini B	65148	6	0	0	1	5	0	0
Kati	78525	9	0	0	1	5	0	0
Kusini	37756	7	0	1	1	5	20	100
Magharibi	462221	8	1	0	1	5	20	0
Mjini	248948	9	0	3	1	5	60	300

*National Bureau of Statistics (2002 census), Population projection 2012

^a^A product of multiplication minimum number of comprehensive required by 5

### Geographic distribution of EmONC facilities

**Fig. 2:**
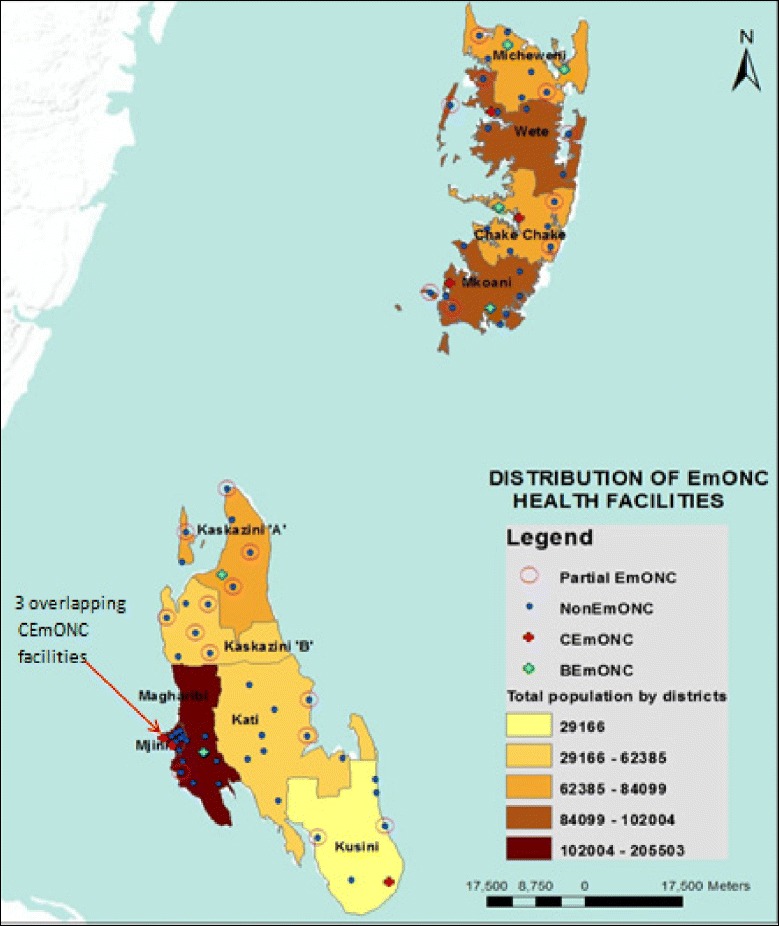
District Populations and distribution of EmONC facilities in Zanzibar, 2012

The distribution of BEmONC and CEmONC varied across Zanzibar. Basic EmONC facilities were mainly available in North Pemba and South Pemba regions, as well as West Urban regions, as shown in Fig. 2. CEmONC facilities were mainly concentrated in Urban West (Unguja); North Pemba and South Pemba regions.

### Proportion of all births in EmONC facilities

Overall, 47 % (25,560/54,057) of all births occurred in EmONC health facilities. The proportion of health facility delivery was slightly higher in Unguja zone as compared to Pemba. The highest proportion of births in health facilities were noted in Chake Chake and South Unguja. South District had the highest proportion of births occurring in EmONC facilities followed by Chake Chake. North B District had a higher proportion of births occurring in non-EmONC facilities as compared to other districts. In the urban districts, women from the outside the catchment areas were often delivering at Mnazi Mmoja National Hospital (MMNH). There were few deliveries occurring in health facilities in Central and West districts and neither had functioning EmONC facilities.

### Met need for EmONC services

According to the WHO handbook on monitoring emergency obstetric care, ‘Met need’ estimates the proportion of all women with major direct obstetric complications who are treated in a health facility providing EmONC services [22]. The international minimum standard is 100 %. Findings from this study indicated that the national met need for EmONC was only 33.1 % (2519/7354) in the 12 months preceding the survey. Unguja zone had a higher met need of 33.7 % (1396/4137) as compared to Pemba zone 22.7 % (930/4100). Urban District had the highest met need at 55.2 % (1315/2383) (Table 3).

**Table 3:** Met need for EmONC

	All HF % (n/N)	EmOC % (n/N)	Non-EmOC % (n/N)
OVERALL	34.3 (2519/7354)	33.1 (2435/7354)	1.1 (84/7354)
Zone			
Unguja	37.2 (1537/4137)	33.7 (1396/4137)	3.4 (141/4137)
Pemba	24.0 (982/4100)	22.7 (930/4100)	1.3 (52/4100)
District			
ChakeChake	15.8 (319/2016)	15.4 (310/2016)	0.4 (9/2016)
Mkoani	5.8 (116/2016)	4.7 (95/2016)	1.0 (21/2016)
Wete	11.5 (239/2085)	11.3 (236/2085)	0.1 (3/2085)
Micheweni	14.8 (308/2085)	13.9 (289/2085)	0.9 (19/2085)
North A	9.2 (112/1220)	8.9 (109/1220)	0.2 (3/1220)
North B	0.6 (7/1220)	0 (0/1220)	0.6 (7/1220)
Central	0.7 (4/535)	0 (0/535)	0.7 (4/535)
South	71/535 (13.3)	11.8 (63/535)	1.5 (8/535)
West	1.1 (26/2383)	0.8 (18/2383)	0.3 (8/2383)
Urban	1317/2383 (55.3)	55.2 (1315/2383)	0.1 (2/2383)

*n* = number of women treated for direct obstetric complications

*N* = expected number of women who would have major obstetric complications

### Caesarean deliveries as a percentage of all births

This indicator calculates caesarean deliveries as a proportion of expected live births in the population in a specified period [18]. It is a measure of accessibility and utilization of critical services [22]. While there are debates about optimal rates, the EmONC indicators recommend a rate between 5 and 15 %. In the 12 months preceding the survey, 4.4 % of expected births in Zanzibar were cesarean deliveries. Unguja zone had a slightly higher caesarean delivery rate as compared to Pemba. The highest caesarean rate was in Urban District at 23.9 %.

### Direct obstetric Case Fatality Rate (CFR)

This indicator provides an indication of quality of care in health facilities. According to the WHO handbook on monitoring emergency obstetric care the maximum acceptable level of CFR is less than one percent [22]. Although there was substantial geographical variation between the districts, the overall direct obstetric case fatality rate was 2.5 % for all health facilities surveyed and 2.2 % in EmONC facilities in the 12 months preceding the survey. The highest CFRs were noted in Wete followed Mkoani and Chake Chake districts in Pemba Island.

### Intrapartum and very early neonatal death

Quality of intrapartum and newborn care was also assessed through facility-based perinatal mortality. The WHO handbook on monitoring emergency obstetric care defines the intrapartum and very early neonatal death rate as the proportion of births that result in a very early neonatal death or an intrapartum death (fresh stillbirth) in an EmONC facility. The numerator for this indicator is the sum of intrapartum and very early neonatal deaths within the first 24 h of life and the denominator was all deliveries in the facilities during the same period [22]. The intrapartum and very early neonatal death rate was 2 % (*n* = 553) in all health facilities visited in the 12 months preceding the survey. The district variation was noted for this indicator, the highest being in South and Urban districts with 5.8 % (*n* = 46) and 2.4 % (*n* = 377) respectively.

### Availability of 24-hour daily services

Health facilities’ in-charges were asked if the facilities provide obstetric and neonatal care services 24 h a day and 7 days a week. Of the visited health facilities, only 18 % (14/79) provide obstetric and neonatal care continuously on a daily basis.

### Cesarean Review

Reviews of three randomly selected Cesarean deliveries from birth registers performed in the last 12 months were conducted at seven health facilities offering Cesarean delivery services, with a total of 21 cases analyzed. Eighteen cesarean cases were classified in the register as emergency and two were elective. The main documented reasons for cesarean delivery were obstructed labour (4 cases), followed by fetal distress (3 cases). However, there was no documentation of the indications for most (14 cases) of the caesarean deliveries reviewed. With regard to the timely management, on average, 67 min elapsed from a decision to the actual performance of cesarean section. The minimum time taken was 30 min and the maximum was 86 min in Mwembeladu and district hospitals respectively. About half (10 cases) of cesarean deliveries were performed by obstetrician/gynecologists, mainly based in the district and private hospitals, while 7 cesareans were conducted by trained Assistant Medical Officers (AMOs) and 4 cases by Medical doctors (MDs). AMOs receive training on emergency obstetric and neonatal care during their pre-service training, but also undergo special three-month competency-based training on cesarean sections and provision of quality EmONC services, while MDs are trained during their pre-service study period and internship.

### Availability of qualified staff to perform EmONC services

The provision of health services depend to a large extent, on the availability of both human resources and properly equipped health facilities. The study found that there was limited supply of human resources in all visited health facilities, particularly for the higher cadres, in relation to Zanzibar minimum staff requirements For example, at MMNH there was a 50 % gap between the planned versus actual number of medical doctors. The met need for nurses (excluding midwives) was only 22.7 %. However, the planned need of midwives surpassed the required number in most of the visited health facilities. At district level, the human resource situation was similar to that in Mnazi Mmoja Hospital. Medical doctors and general surgeons were not available at any of the district hospitals where six are required by policy. Obstretrician-Gynecologists were only available 33.3 % of staffing establishment at the time of the survey. With regard to the availability of trained AMOs, the study found that only 10.0 % of the AMOs and 11.1 % of anesthesiologists were available at PHCCs. There was also insufficient numbers of the most important cadres to perform duties at PHCU+ level. At private hospitals the availability of human resources was quite similar with exception of greater availability of MDs and Anesthesiologists (MD).

### Infrastructure, communication, drugs, equipment, and supplies

The survey team inspected the infrastructure of 35 health facilities offering delivery services to see if they were convenient for both staff and patients. Overall, 91 % (32/35) of the facilities had good infrastructure and nearly all had sufficient light source to perform tasks during the day and night. More than 85 % of health facilities’ toilets were in good condition and well-functioning. Water was available in 86 % (30/35) of the visited facilities offering delivery services. Privacy was well guaranteed with presence of curtains in 86 % (30/35) of facilities.

With regard to communication, the study found that 30.4 % (24/79) of all the visited health facilities had a functional mobile phone owned by the facility. Every health facility had individual health providers who had personal, functional mobile phones and 78.5 % (62/79) of the staffs used their own mobile phones to refer patients to the next level of care.

Oxytocics and prostaglandins drugs were available at 39 % (31/79) of all health facilities visited. Among those facilities reported to have oxytocics and prostaglandins, oxytocin was highly available at 90 % (28/31) followed by misoprostol at 71 % (22/31). Anticonvulsants drugs were available at 43 % (34/79) in visited health facilities.

Overall, general equipment and supplies needed for labor and delivery services were available at 60 % (21/35) of facilities offering delivery services. Fetal stethoscope and kidney basins were equally available at 97.1 % (34/35). The supplies which were reasonably found in all health facilities offering delivery services included: IV cannulae 71.4 % (25/35), clinical oral thermometer 68.6 % (24/35), suture needles/suture materials 65.7 % (23/35) and examination table 77.1 % (27/35). Labor/delivery tables with and without stirrups were available at 45.7 % (16/35). Partographs were available at 51.4 % (18/35) while adult ventilator bags and mask were available at 25.7 % (9/35). Ultrasounds were available at 23 % (8/35).

## Discussion

For every 500,000 population, the minimum acceptable level is five EmONC facilities with at least one of which provides comprehensive care [22]. This study has shown that the minimum acceptable level of EmONC services in Zanzibar overall has been met, although, urban district facilities are overloaded with deliveries which poses rising concerns regarding ability to provide high quality care. While the minimum has been met overall, distribution of facilities is not equitable. The assessment revealed that there was a gap of 50 % of EmONC facilities in Unguja, while Pemba zone surpassed the minimum by 40 %. Most of the CEmONC services were largely found in the urban districts, raising concern about accessibility for some parts of the islands. Similar findings have been reported in other low-income countries [18, 24]. Furthermore, in some situations where the population is widely dispersed and travel is difficult; EmONC facilities will likely need to exceed the minimum acceptable level for more equitable geographic distribution [22].

Although most facility-based deliveries are taking place in EmONC health facilities, few deliveries were noted in Central, North B and West districts. Furthermore, the study found that there was a high volume of deliveries in the urban district. These deliveries might have been conducted in lower health facilities like PHCU + s and PHCCs, hence reducing the workload at tertiary level. Human resource and infrastructural capacity would be needed to ensure quality delivery services at lower level health facilities. Unlike Unguja, Pemba has a relatively even distribution of EmONC facilities; however, most of these EmONC facilities are underutilized for delivery services. Specialists perform most of the caesarean sections as compared to the trained AMO’s. Yet, most of these Caesarean sections are performed in the district and private hospitals. Ideally, specialists at MMNH referral hospital should have attended most of the complicated cases but this was not the case. In comparison, to the mainland, 80 % of Caesarean sections are performed by trained AMO’s [25, 26].

Met need for EmONC is another indicator that reflects a health system’s capacity to respond to women’s obstetric needs when complications develop [18]. The met need has reached only half the acceptable level, meaning that a large proportion of women who experienced complications did not receive the medical care they needed.

The majority of the caesarean sections were for emergencies, which show that these occur at a large extent due to (assumed) poor monitoring of pregnancies, given the types of ‘expected’ common complications. In fact, the issue of delays corroborates these findings as specialists are few but also it seems that even AMOs are too few on the islands. Generally, human resource data found in the survey suggests that there is a gap of specialists in CEmONC health facilities in particular among the higher cadres.

Although the WHO handbook on monitoring emergency obstetric care recommends 5 %–15 % of pregnancies are expected to require Cesarean delivery [22], what matters most is for all women who need this service to receive it. The highest caesarean rate (23.9 %) in Urban district is worrying and needs a careful interpretation. Most of these caesareans are happening at MMNH, which is a referral hospital that serves all six districts in Unguja zone. However, if the caesarean rate is included in the overall served six districts of Unguja zone, it is reduced to 3.6 %.

The maximum acceptable level of direct obstetric case fatality rate can sometimes be misinterpreted due to the lack of comparability among health facilities. For example, women with the most serious complications may be referred from the PHCCs and die at MMNH referral hospital, hence increasing the direct obstetric case fatality rate in the hospital. The high overall CFR suggests poor quality of care in the respective health facilities, which may include delayed and inappropriate referrals. Inadequate human resources, limited availability of equipment and supplies might have contributed to the higher CFR. The study did not capture specific reasons for the alarming high CFR in Wete, Mkoani and Chake Chake districts and deeper understanding of quality of care should therefore be sought.

There was also district variation with regard to the intrapartum and very early neonatal deaths, with the highest rates in South, Wete and Urban districts. Poor monitoring of progress of labour and delay in management of complications may be at the root of many of these deaths. Manual vacuum aspiration/dilation and curettage, and AVD were the least performed signal functions in PHCU + s. This reflects both the need for training and improving utilization of services. Similar findings have been reported in other low-income countries [17, 18].

This study has limitations and strengths that should be borne in mind. For instance, if the direct obstetric case fatality rate is acceptable, but EmONC coverage or met need is insufficient, the implication is that women who deliver in EmONC facilities are likely to survive but maternal death outside health facilities might still be common as only half of women deliver in facilities [22]. Hence, community studies are important to explore the reasons for underutilization of healthcare services. In general, the number of deliveries reviewed here were few; therefore the percentages should be interpreted with caution. It would be useful to learn from health care providers in the facilities as to why particular signal functions are not performed. The interpretation of caesarean section review should also be taken with precaution as the very low number of the reviews meant that findings could not be expressed in percentages. The small sample was a result of the low numbers of health facilities providing caesarean deliveries in Zanzibar. Additionally, we noted that there was inadequate documentation of obstetric emergencies in the delivery record books, which may partially explain the low met need for EmONC. For example, fresh stillbirths are quite often documented as macerated stillbirths and obstetric emergencies are sometimes not indicated in the delivery record books especially after treatment. Thus, despite these potential concerns, our findings can inform the development of intervention strategies aimed at saving the lives of mothers and newborn babies.

## Conclusions

Despite Zanzibar having met the minimum standards for EmONC availability per population, the met need is still quite low. With only 50 % delivering in health facilities it will be very hard to reach this without increase in utilization. Evidence about quality of care and availability of human resources and supplies, as well as geographic distribution, might explain why these numbers are still low. Yet it will be essential to also make sure that women seek care in order to make real change in this coverage. In order to improve quality of care it is important to consider other important issues like effective training, and monitor the success through supportive supervision and good leadership by senior physicians, which may also encourage women to use services. The use of evidence-based practices should highly be encouraged at all levels. Audit within medical institutions are needed to review patient charts and determine whether the indications for cesarean delivery are appropriate and to determine the causes behind the high case fatality rates found in this survey [27].

## Abbreviations

AMDD, Averting Maternal Death and Disability; AMOs, Assistant Medical Officers; AVD, Assisted Vaginal Delivery; BEmONC, Basic Emergency Obstetric Neonatal Care; CeMONC, Comprehensive Emergency Obstetric Neonatal Care; CFR, case fatality rate; DANIDA, Danish International Development Agency; MCH, Maternal and child health; MD, Medical doctor; MDGs, Millennium Development Goals; MMNH, Mnazi Mmoja National Hospital; MRP, manual removal of the placenta; PHCC, Primary Health Care Centers; PHCUs, Primary Health Care Units; RA, Research Assistance; TDHS, Tanzania Demographic Health Survey; UN, United Nations; WHO, World Health Organisation; ZMOH, Zanzibar Ministry of Health.
